# Examining the Impact of Young Children’s Motor Development on Inhibitory Control and Social Skills

**DOI:** 10.3390/children13030318

**Published:** 2026-02-25

**Authors:** Ali Brian, Shea E. Ferguson, Angela Starrett, Emily Kallis, J. Ross Ramsey

**Affiliations:** 1 Department of Educational and Developmental Sciences, College of Education Columbia, University of South Carolina, West Columbia, SC 29172, USA; sferguson@sc.edu (S.E.F.); starrett@mailbox.sc.edu (A.S.); ekallis@email.sc.edu (E.K.); 2 Belk Center for Community College Leadership and Research, North Carolina State University, Raleigh, NC 27603, USA; jrramse2@ncsu.edu

**Keywords:** manipulative skill, ball skill, executive function, early childhood, social-emotional

## Abstract

**Background/Objectives:** Understanding how motor and cognitive development may contribute to prosocial behavior is essential for supporting whole-child development in early learning settings. Early childhood education settings are well-positioned to address these concerns through integrated approaches that support the whole child. Research exploring the mechanisms that link developmental domains remains limited, especially regarding how motor development may influence social outcomes through cognitive processes such as inhibitory control. Thus, the purpose of this study was to examine whether gains in motor skills were associated with prosocial behaviors indirectly through improvements in inhibitory control. **Methods:** Preschoolers (*N* = 238; *M_age_* = 66.61, *SD* = 9.06 months; Girls = 45%) participated in a dual-component intervention supporting motor and social-emotional development. **Results:** For boys, growth in object-control skills predicted gains in inhibitory control, which in turn predicted both self-control and cooperation. For girls, object-control skills were not significantly related to inhibitory control, although inhibitory control was positively related to self-control. **Conclusions:** Findings highlight the interconnectedness of motor, cognitive, and social development in early childhood and the potential for interventions to yield cross-domain benefits, especially for boys. Engaging in motor skill activities, particularly object control, may foster both inhibitory control and prosocial behaviors. Programs should intentionally create inclusive environments that encourage girls’ engagement in object-control tasks and boys’ inhibitory control, thereby promoting equitable developmental opportunities across domains.

## 1. Introduction

Early childhood is a critical period for the learning and development of motor competence, executive functioning, and prosocial behavior [1,2,3]. During this stage, children learn and develop goal-directed movements known as fundamental motor skills, which serve as the starting block for more complex physical activity and are essential for engaging in peer-based play [4]. These fundamental motor skills include both locomotor movements (e.g., walking, jumping) and object-control skills (e.g., catching, kicking) [5]. At the same time, central cognitive processes such as working memory, cognitive flexibility, and inhibitory control, collectively referred to as executive functions, are developing [6,7,8]. Inhibitory control, a core component of executive function, refers to the ability to suppress automatic, impulsive, or goal-inappropriate actions [9], enabling children to pause before acting, resist distractions, and respond in socially appropriate ways [10,11].

### 1.1. Interactions Among Motor, Cognitive, and Social Development

Motor, cognitive, and social domains are dynamically interconnected, with advances in one domain often influencing development in another [2,12,13]. Motor experiences that require planning, coordination, and behavioral adjustment may simultaneously engage executive processes and social behaviors [12,14,15]. These links are particularly evident in socially embedded motor contexts such as group games and peer play, where cooperation and behavioral regulation are required. Executive functions, particularly inhibitory control, may represent a central mechanism through which motor competence contributes to prosocial development [10]. Clarifying these pathways is essential for informing integrated approaches to early education and intervention [12,16].

### 1.2. Motor Development and Its Role in Cognitive and Social Development

Object-control skills require children to coordinate hand–eye movements, plan sequences of actions, and adjust their behavior in response to external feedback [17]. These demands engage neural systems, most notably the prefrontal cortex and cerebellum, which are also important for engaging in executive functions [15,18]. For example, the cerebellum, traditionally associated with motor coordination, also contributes to cognitive timing, pattern recognition, and inhibition. Furthermore, object-control tasks often require children to inhibit impulsive movements, follow sequential rules, and make split-second adjustments; therefore, they may serve as a functional context for practicing and refining inhibitory control skills [19,20,21]. Thus, children who show greater gains in object control also demonstrate stronger inhibitory control [17,22].

Beyond cognitive connections, object-control skills may also support social development by enabling more frequent and confident participation in peer play [23,24]. Physical activities such as throwing, catching, or kicking often occur in group settings and require cooperation and self-control [23]. When children feel competent in object-control skills, they may be more likely to initiate or engage in active peer play, providing opportunities to practice and reinforce social-emotional skills [25,26]. Subsequently, peer play may initiate a positive feedback loop where motor competence leads to greater social engagement, which in turn reinforces motor and social development [26].

### 1.3. Inhibitory Control as a Bridge

Inhibitory control may serve as the bridge between motor competence and prosocial behavior. Inhibitory control plays a foundational role in prosocial behavior by enabling children to regulate their emotions and actions in socially appropriate ways [27,28]. Inhibitory control facilitates cooperation, conflict resolution, and delayed gratification [29,30]. For example, children who are better able to pause before reacting are more likely to comply with group norms and respond sensitively to peers [31]. These inhibitory skills are essential for cooperation and self-control [32]. Stronger inhibitory control skills in children are related to having greater emotional understanding, positive peer relationships, and displaying less disruptive behaviors [11,33,34,35]. Therefore, inhibitory control may serve as a key mechanism through which early motor development influences prosocial outcomes [36].

### 1.4. Differences by Biological Sex

There is growing recognition that boys and girls may experience these above-mentioned developmental trajectories differently. For example, boys tend to enter preschool with lower levels of prosocial skills but may show greater gains over time [37]. Similarly, boys and girls may engage in inhibitory control differently [38]. On tasks requiring inhibition, boys often responded faster but made more errors, while girls tended to be more cautious and accurate [39,40]. These differential patterns suggest that girls may utilize inhibitory control more consistently during early childhood, while boys may exhibit steeper gains over time. Furthermore, this effect could be stronger in boys plausibly due to early hormonal influences, particularly testosterone, which promote neurogenesis, synaptic remodeling, and maturation of motor and executive function networks [41,42], warranting further exploration.

### 1.5. Current Study

Despite the recognized importance of motor, cognitive, and social-emotional development in early childhood, there are secular declines in preschoolers’ motor development, physical activity, and social-emotional skills [43,44,45,46]. Early childhood education settings are well-positioned to address these concerns through integrated approaches that support the whole child [17,47,48]. However, research exploring the mechanisms that link developmental domains remains limited, especially regarding how motor development may influence social outcomes through cognitive processes such as inhibitory control [36]. Moreover, researchers have called for intervention research to examine directionality and identify conditions that strengthen these trajectories [17,36,49].

Within the present study, we addressed these gaps through a secondary analysis of data from a dual-component classroom-based intervention (see [16]). We tested whether gains in object-control skills among preschool-aged children predicted self-regulation and prosocial behavior, and whether these impacts were mediated by inhibitory control. We focused specifically on the intervention group as this allows us to isolate and examine theoretically grounded pathways of change within the context of a targeted program designed to support both motor and behavioral development via the following three research questions. First, what is the direct effect of object-control skill development on inhibitory control, cooperation, and self-control? Second, what is the indirect effect of inhibitory control on the relationship between object-control skills and cooperation and object-control skills and self-control? Third, do these relationships differ by sex? Based on prior literature (e.g., [17,36]), we hypothesized that object-control skills would positively relate to both inhibitory control and prosocial skills, and inhibitory control would have an indirect effect on this relationship, with a stronger effect for boys (e.g., [41,42]).

## 2. Materials and Methods

### 2.1. Design, Participants, and Setting

This study features a secondary analysis of data from the intervention arm of a larger trial (see [16]), using a single-group pre-post design. All children in the current study received the full intervention. Participants included preschoolers (*N* = 238; *M_age_* = 66.61, *SD* = 9.06 months; Girls = 45%) enrolled at a large rural early childhood center (ECC) in the southeastern United States. In terms of race and ethnicity, 71% of children identified as Non-Hispanic White, 23% as Black, 2% as Hispanic, 0.4% as Asian, and 3.6% as another race or ethnicity.

The ECC comprised approximately 32 classrooms and maintained an average annual enrollment of 533 students, with a demographic profile of 50% female and a racial/ethnic distribution of 61% Non-Hispanic White, 27% Black, 5% Hispanic, 0.4% Asian, and 6.6% other. All enrolled children qualified for free or reduced lunch, and 85% lived in poverty, as defined by enrollment in Medicaid, Temporary Assistance for Needy Families (TANF), or the Supplemental Nutrition Assistance Program (SNAP).

### 2.2. Intervention

The intervention combined two established programs: (a) PAX Good Behavior Game (PAX-GBG), a classroom-based positive behavior program [50], and (b) Successful Kinesthetic Instruction for Preschoolers (SKIP), a gross motor skill intervention [14,51]. Both components were implemented during physical education classes, with PAX-GBG also incorporated into general education classroom activities.

#### 2.2.1. PAX-GBG

PAX-GBG is grounded in the Caring Classroom Environment Framework and uses a set of evidence-based strategies, known as “PAX kernels”, to reinforce prosocial behaviors and self-regulation skills. These kernels include prompts and routines such as PAX Leader, PAX Quiet, PAX Voices, PAX Vision, Beat the Timer, and PAX Hands and Feet, each serving as a cue to help children focus on specific positive behaviors. Prior research with older students has shown that PAX-GBG can increase prosocial behavior, improve academic performance, reduce disruptive behaviors [52,53], and support teacher well-being and self-efficacy [54].

The dual-component intervention was the first known application of PAX-GBG in an early childhood center setting with children aged 3–6 years. To suit developmental needs, the standard protocol was adapted while maintaining all 13 core kernels. Each lesson or game emphasized no more than three kernels, and the “PAX Games” used for practice were shortened from the typical 5 min to 2.5–5 min. These adjustments allowed children to gradually build competence in specific behaviors, such as keeping their hands to themselves and using appropriate voice levels.

#### 2.2.2. SKIP

SKIP is a structured motor skill intervention that promotes the acquisition of fundamental motor skills through a process-oriented teaching approach. The program provides explicit instruction, demonstration, and guided practice of fundamental motor skills in a supportive environment that offers multiple opportunities for skill repetition. Activities are developmentally appropriate and designed to improve locomotor and object-control skills. SKIP has consistently produced large improvements in fundamental motor skills across diverse groups of implementers, including researchers, physical education teachers, classroom teachers, and parents [14,51].

### 2.3. Instrumentation

#### 2.3.1. Object-Control Skills

The Test of Gross Motor Development–Third Edition (TGMD-3; [55]) is a standardized motor assessment for children ages 3 to 10 years, 11 months that can be scored using normative or criterion-referenced methods, with strong psychometric properties [56]. Only the object-control subscale was used in this current study. The object-control subscale includes seven skills: one-hand stationary dribble, two-hand strike of a stationary ball, one-hand forehand strike of a self-bounced ball, underhand throw, kick a stationary ball, two-hand catch, and overhand throw.

Each skill includes three to five process-oriented criteria scored dichotomously (1 = observed, 0 = not observed). After a demonstration by the assessor, children complete one practice trial. If a different skill is performed (e.g., kicking the ball during dribbling), the skill is re-demonstrated before repeating the practice trial. Two scored performance trials are then completed for each skill. Possible points per skill range from 6 to 10, with a total maximum score of 54 for the object-control subscale.

#### 2.3.2. Prosocial Skills

The Social Skills Improvement System rating scale (SSIS; [57]) was used to measure prosocial skills, specifically, cooperation and self-control. Six items assessed cooperation within the classroom environment (e.g., “Follows your directions”, “Ignores classmates when they are distracting”, α = 0.89) and seven items assessed self-control (e.g., “Uses appropriate language when upset”, “Makes a compromise during a conflict”, α = 0.89). Classroom teachers utilized the SSIS computer entry form that includes a paper book and were asked to indicate how often each child exhibits a specific behavior on a four-point scale (never [0], seldom [1], often [2], and almost always [3]). Raw scores were averaged, and higher scores indicated higher cooperation and self-control skills. Overall, test–retest reliability is *r* = 0.81–0.84 [57].

#### 2.3.3. Inhibitory Control

The 3–7-year-old Flanker Inhibitory Control and Attention Test from the NIH Toolbox Early Childhood Cognition Battery [58] was used to assess inhibitory control and visual attention. The task presents a central arrow flanked by two arrows on each side, and children are asked to indicate the direction of the central arrow by pressing the corresponding on-screen button. Trials alternate between congruent conditions, where flankers point in the same direction, and incongruent conditions, where flankers point in the opposite direction. The scoring algorithm integrates both accuracy and reaction time. There is no fixed scoring range for the Flanker task, as reaction time has no upper limit; thus, there is no predetermined scoring range. The Flanker task has demonstrated strong reliability in preschool-aged children, with test–retest reliability of 0.85 and an intraclass correlation coefficient of 0.83 [58].

### 2.4. Procedures

This study featured a secondary data analysis drawn from a larger project that originally included both intervention and control participants. However, the current analysis focuses exclusively on children who received the intervention. Data collection for the original project spanned three academic years, from 2020–2021 to 2022–2023 (see [16]). All procedures were approved by the University of South Carolina Institutional Review Board (PRO00091603) at the lead author’s institution. Recruitment was inclusive, inviting all families with children enrolled at the ECC to participate. Eligibility required attendance at least one ECC school-based session. Parents provided written informed consent, and children gave verbal assent prior to participation.

Two weeks before the intervention began, trained research staff administered the TGMD-3 on-site at the ECC, following standardized protocols. All TGMD-3 trials were video recorded and coded by trained staff who demonstrated strong reliability. Coders were trained by an expert with documented accuracy exceeding 98% and were required to achieve at least 85% inter-rater agreement with the expert before coding study data. Intra-rater reliability was maintained at or above 90% throughout the study. To ensure consistency, inter-rater reliability with the expert was assessed on 30% of the sample at both baseline and posttest, with a minimum of 90% agreement required. All videos were coded by assessors blinded to timepoint and group assignment, and coders were randomly assigned videos after confirming reliability on a subset of recordings.

After pretesting, physical education teachers trained in the SKIP program implemented SKIP twice weekly for nine months during regular school hours. Sessions lasted 30 min and took place in the ECC gymnasium during two of the children’s daily recess blocks. On non-SKIP days, children participated in their usual recess activities. Concurrently, classroom teachers implemented the PAX Good Behavior Game (PAX-GBG) daily, integrating its strategies into routine classroom activities. Teachers received training prior to implementation.

To monitor fidelity, all SKIP lessons were digitally recorded. A member of the research team randomly selected and coded 30% of the recorded lessons using a standardized SKIP Fidelity Checklist [14]. Physical education teachers maintained fidelity levels between 85% and 100% throughout the intervention. For PAX-GBG, classroom teachers completed daily logs, and research staff conducted weekly classroom observations. Fidelity for PAX-GBG implementation was highly variable (see [16] for more details).

Identical TGMD-3 protocols were repeated within two weeks following intervention completion. The classroom teachers completed the Social Skills Improvement System (SSIS) rating scales for each student. Research staff entered all SSIS data using a triple-pass entry protocol. The Flanker task was also administered during the two weeks following the intervention. The Flanker Test (3–7-year-old version) began with a fish-themed practice phase to ensure comprehension. Children who achieved at least 90% accuracy during practice advanced to 20 test trials, which included a pseudorandom mix of congruent and incongruent arrow stimuli. The task was administered on an iPad, with instructions provided both visually and orally. Raw Flanker scores were used in analyses, with age included as a covariate and sex examined as a moderator.

### 2.5. Analysis Plan

All analyses were conducted in Mplus v8.10 using maximum likelihood estimation with robust standard errors (MLR), which provides standard errors and chi-square statistics robust to non-normality. To account for the nested structure of the data (students within classrooms), the TYPE = COMPLEX option was used with the teacher specified as the cluster variable.

A multigroup path model was specified to examine differences by biological sex. The model tested whether posttest object control predicted posttest inhibitory control (Flanker task), which in turn predicted posttest prosocial skills (cooperation, self-control). Direct paths from posttest object control to each prosocial skill were also estimated. Age in months at posttest was included as a covariate for all endogenous variables. Baseline object-control scores were included as a covariate for posttest object control; baseline scores for other outcomes were not available and thus not included.

Indirect effects from posttest object control to each prosocial skill via inhibitory control were estimated. Missing data were handled using full information maximum likelihood under the missing-at-random assumption. Model fit was examined using the following indices: chi-square value (χ^2^), root mean square error of approximation (RMSEA), comparative fit index (CFI), Tucker–Lewis Index (TLI), and standardized root mean square residual (SRMR). Adequate model fit was assessed via nonsignificant χ^2^ values (*p* > 0.05), CFI and TLI > 0.90, RMSEA < 0.08, SRMR < 0.10 [59,60]. Direct effects were interpreted using both unstandardized (*b*) and standardized (β) estimates. Indirect effects were evaluated using 95% bootstrapped bias-corrected confidence intervals with 500 bootstraps. To evaluate how well the model accounted for variability in endogenous variables, *R*^2^ values were assessed.

## 3. Results

### 3.1. Model Fit

Since χ^2^-based fit indices are not available for TYPE=COMPLEX models in Mplus, model fit was evaluated using a version of the model that ignored classroom clustering. These results, except for SRMR, represent the upper bound for possible fit. The multigroup path model controlling for age demonstrated adequate fit: *χ*^2^(10) = 15.38, *p* = 0.12, CFI = 0.98, TLI = 0.95, RMSEA = 0.067, 90% CI [0.00, 0.13], SRMR = 0.053. All fit indices met or exceeded conventional benchmarks for acceptable model fit [59,60].

### 3.2. Direct Effects

Cluster-adjusted standardized and unstandardized path estimates are shown in Table 1. For both boys and girls, posttest object-control skills were strongly predicted by baseline object-control skills, with boys demonstrating slightly higher growth (boys: b = 0.46, SE = 0.09, β = 0.41, 95% CI [0.34, 0.55]; girls: b = 0.40, SE = 0.10, β = 0.36, 95% CI [0.21, 0.51]).

Among boys, posttest object-control skills were positively associated with inhibitory control (b = 0.36, SE = 0.16, β = 0.30, 95% CI [0.11, 0.52]). Inhibitory control, in turn, was positively associated with both self-control (b = 0.14, SE = 0.05, β = 0.40, 95% CI [0.16, 0.69]) and cooperation (b = 0.09, SE = 0.03, β = 0.30, 95% CI [0.14, 0.55]).

For girls, posttest object-control skills did not significantly predict inhibitory control (b = 0.09, SE = 0.27, β = 0.05, 95% CI [−0.24, 0.36]). However, inhibitory control was positively associated with self-control (b = 0.11, SE = 0.04, β = 0.33, 95% CI [0.06, 0.59]). No statistically significant direct effect was found from inhibitory control to cooperation (b = 0.08, SE = 0.04, β = 0.28, 95% CI [−0.01, 0.58]).

### 3.3. Indirect Effects

**Figure 1:**
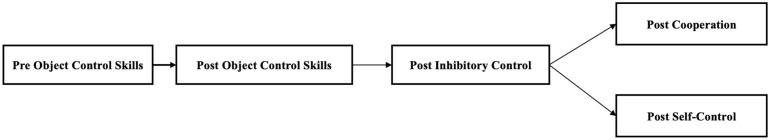
Final Structural Model for Girls and Boys. **Note**. For simplicity, the covariate of children’s age was not included in the figure.

The indirect effect of inhibitory control on the relationship between posttest object-control skills and prosocial skills was examined (see Figure 1). For boys, there was a statistically significant indirect effect of object-control skills on cooperation (b = 0.032, 95% CI [.009, 0.070]) and on self-control (b = 0.048, 95% CI [.011, 0.119]) through inhibitory control. These findings suggest that improvements in motor skills may strengthen executive function, which in turn supports prosocial behaviors. For girls, neither indirect effect was significant (cooperation: b = 0.007, 95% CI [−0.039, 0.070]; self-control: b = 0.010, 95% CI [−0.045, 0.066]).

### 3.4. Proportion of Variance

The model accounted for a larger proportion of variance in boys’ outcomes, particularly for inhibitory control and prosocial skills, relative to girls. For boys, R^2^ values indicated that the model explained 31.5% of the variance in posttest object control, 17.9% in inhibitory control, 14.1% in self-control, and 10.0% in cooperation. For girls, the model explained 28.0% of the variance in posttest object control, 13.2% in inhibitory control, 9.8% in self-control, and 7.7% in cooperation (Table 2).

## 4. Discussion

Findings from this study provide partial support for our hypothesis that gains in motor skills can be indirectly associated with social behaviors through gains in inhibitory control. For boys, growth in object-control skills was associated with improvements in inhibitory control, which, in turn, predicted both self-control and cooperation. For girls, object control was not significantly related to inhibitory control, although inhibitory control itself was positively associated with self-control. These results align with prior work emphasizing the central role of executive function in helping children manage impulses, cooperate with peers, and adhere to social norms [35].

The proportion of variance explained by the model offers meaningful insight into the developmental pathways linking motor competence, inhibitory control, and prosocial behavior. As hypothesized, the model accounted for more variance in inhibitory control and prosocial skills for boys than for girls, suggesting that the path from motor to cognitive to social domains may be more pronounced in male children. This pattern aligns with findings that boys tend to exhibit stronger object-control skills in early childhood, which are associated with increased engagement in physically active and competitive play that supports executive functioning [61]. Boys also show higher levels of externalizing behaviors, such as aggression and impulsivity, making inhibitory control a particularly critical mediator for their social development [62]. In contrast, girls often demonstrate stronger early social motivation and prosocial tendencies, which may reduce their reliance on motor-driven pathways to develop social competence [63]. Compounded with our earlier discussion of hormonal influences, neurodevelopmental variations influenced by early hormonal exposure, particularly elevated testosterone levels in boys, may contribute to sex-specific patterns of brain maturation. These hormonal influences enhance synaptic growth in motor and prefrontal regions and may amplify the developmental impact of motor competence [41,42]. However, these perspectives are offered here solely as a broader interpretive framework for the observed patterns. Nonetheless, consideration of biological and behavioral sex differences may provide useful context for understanding variability in associations between motor competence and cognitive or social outcomes. These considerations may help inform the design of future research and intervention strategies that explicitly examine these processes.

### Strengths and Limitations

Despite the strengths of this study, several limitations warrant acknowledgement. First, participants were from a single early childhood center in a rural southeastern U.S. region, which may limit the generalizability of the findings to broader populations. Second, the measure of inhibitory control relied on a single computerized task (NIH Flanker), which, although psychometrically sound, may not fully capture the multidimensional nature of executive functioning or reflect real-world regulation (e.g., [64]). Third, although sex differences were examined through multigroup analysis, the study may not have been adequately powered to detect subtle subgroup differences. While this study focused on the paths initiated by motor skill growth, these gains occurred in the context of a dual-component intervention designed to support both physical and social-emotional development. Plausibly, the PAX-GBG component provided complementary supports that reinforced children’s emerging inhibitory control and prosocial behavior. Future research could explore a multi-arm trial disentangling the intervention and gaining awareness of the individual contributions of SKIP and the PAX-GBG, as well as the potential for a multiplicative effect of the package program. Additionally, future studies should explore potential delayed or longitudinal effects of motor-based interventions, particularly for girls (e.g., replicate [65]), and examine whether inhibitory control consistently serves as an indirect mechanism across time. Furthermore, designs that incorporate multiple, ecologically valid measures of executive function (e.g., [64]) and social behavior would further strengthen the field’s understanding of these developmental pathways. Next, examining the impact or differential effects of the environment (such as rural vs. urban, etc.) could enhance the variance explained in future studies. For example, Liao and colleagues found that improvements with physical development were moderated by aspects of the preschool environment (e.g., availability of equipment, outdoor space, teacher effects, etc.) [49]. Thus, expanding those data to also include our hypotheses regarding cognitive and social development would be a fruitful and impactful next step. Finally, although the path model specifies directional relationships, the observational nature of the data precludes causal inference. The present study does not directly assess hormonal or neurobiological processes, and the absence of baseline measurements and the single-group design substantially limit inferences regarding developmental change or causality. Accordingly, the results should be interpreted with caution, as patterns of association rather than evidence of underlying biological mechanisms.

Overall, findings from this study highlight the interconnectedness of motor, cognitive, and social development in early childhood, suggesting that interventions targeting one domain may yield cross-domain benefits. However, the pathways observed were more pronounced for boys than for girls, underscoring the need to consider timing, structure, and content when designing early interventions. Early childhood programs may be most effective when they embed motor skill development within socially rich contexts (e.g., peer play and cooperative games) that simultaneously foster executive functions and prosocial behaviors. By attending to these developmental interdependencies, whole-child approaches to early education can provide a stronger foundation for children’s long-term adjustment, well-being, and success.

## Figures and Tables

**Table 1:** Standardized Path Coefficients for Girls and Boys.

Pathways		Boys				Girls			
Outcome	Predictor	*b*	*SE*	β	*p*	*b*	*SE*	β	*p*
Post-Object-Control	Age	0.246	0.071	0.228	<0.001	0.199	0.079	0.254	0.011
Post-Object-Control	Pre-Object-Control	0.455	0.094	0.412	<0.001	0.404	0.103	0.362	<0.001
Inhibitory Control	Age	0.245	0.145	0.191	0.091	0.474	0.242	0.339	0.050
Inhibitory Control	Post-Object-Control	0.360	0.159	0.304	0.024	0.090	0.265	0.050	0.735
Cooperation	Age	0.015	0.045	0.040	0.738	−0.001	0.048	−0.003	0.980
Cooperation	Inhibitory Control	0.088	0.030	0.301	0.003	0.078	0.041	0.278	0.058
Self-Control	Age	−0.038	0.053	-0.088	0.472	−0.024	0.044	−0.051	0.592
Self-Control	Inhibitory Control	0.135	0.051	0.395	0.009	0.109	0.036	0.327	0.002

**Table 2:** Proportion of Variance Explained (R^2^) for Girls and Boys.

Endogenous Variable	Girls *R*^2^	Boys *R*^2^
Posttest Object Control	0.280	0.315
Inhibitory Control	0.132	0.179
Self-Control	0.098	0.141
Cooperation	0.077	0.100

## Data Availability

The raw data supporting the conclusions of this article will be made available by the authors on request.
